# BCL2-Family Dysregulation in B-Cell Malignancies: From Gene Expression Regulation to a Targeted Therapy Biomarker

**DOI:** 10.3389/fonc.2018.00645

**Published:** 2019-01-07

**Authors:** Benoît Tessoulin, Antonin Papin, Patricia Gomez-Bougie, Celine Bellanger, Martine Amiot, Catherine Pellat-Deceunynck, David Chiron

**Affiliations:** ^1^CRCINA, INSERM, CNRS, Université d'Angers, Université de Nantes Nantes, France; ^2^L'Héma-NexT, i-Site NexT Nantes, France; ^3^Department of Hematology, Centre Hospitalier Universitaire Nantes, France; ^4^CNRS GDR3697 Micronit Tours, France

**Keywords:** BCL2, B-cell malignancy, lymphoma, cell death, microenvironment, data mining, predictive markers

## Abstract

BCL2-family proteins have a central role in the mitochondrial apoptosis machinery and their expression is known to be deregulated in many cancer types. Effort in the development of small molecules that selectively target anti-apoptotic members of this family i.e., Bcl-2, Bcl-xL, Mcl-1 recently opened novel therapeutic opportunities. Among these apoptosis-inducing agents, BH3-mimetics (i.e., venetoclax) led to promising preclinical and clinical activity in B cell malignancies. However, several mechanisms of intrinsic or acquired resistance have been described *ex vivo* therefore predictive markers of response as well as mechanism-based combinations have to be designed. In the present study, we analyzed the expression of the BCL2-family genes across 10 mature B cell malignancies through computational normalization of 21 publicly available Affimetrix datasets gathering 1,219 patient samples. To better understand the deregulation of anti- and pro-apoptotic members of the BCL2-family in hematological disorders, we first compared gene expression profiles of malignant B cells to their relative normal control (naïve B cell to plasma cells, *n* = 37). We further assessed BCL2-family expression according to tissue localization i.e., peripheral blood, bone marrow, and lymph node, molecular subgroups or disease status i.e., indolent to aggressive. Across all cancer types, we showed that anti-apoptotic genes are upregulated while pro-apoptotic genes are downregulated when compared to normal counterpart cells. Of interest, our analysis highlighted that, independently of the nature of malignant B cells, the pro-apoptotic BH3-only *BCL2L11* and *PMAIP1* are deeply repressed in tumor niches, suggesting a central role of the microenvironment in their regulation. In addition, we showed selective modulations across molecular subgroups and showed that the BCL2-family expression profile was related to tumor aggressiveness. Finally, by integrating recent data on venetoclax-monotherapy clinical activity with the expression of BCL2-family members involved in the venetoclax response, we determined that the ratio *(BCL2*+*BCL2L11*+*BAX)/BCL2L1* was the strongest predictor of venetoclax response for mature B cell malignancies *in vivo*.

## Introduction

B cell differentiation is a tightly controlled process that leads to the generation and selection of memory B cells and antibody-secreting plasma cells (1, 2). B cells constitute an essential part of our adaptive immune system but the genomic instabilities necessary for the development of high affinity antibodies are also involved in the initiation of malignant B-cell neoplasms (3, 4). Thereby, hematological malignancies can arise from most steps of B cell differentiation and more than 40 types of mature B cell lymphomas are referenced in the latest World Health Organization classification. The most frequent types include diffuse large B cells lymphoma, DLBCL (25%), plasma cell neoplasms [including multiple myeloma, MM (23%)], chronic lymphocytic leukemia, CLL (19%), follicular lymphoma, FL (12%), splenic marginal zone lymphoma, SMZL (7%), mantle cell lymphoma, MCL (3%), hairy cell leukemia, HCL (2%), and Burkitt lymphoma, BL (1%) (5). All of these hematological malignancies are characterized by their own genetic hallmarks, even though most of them display deregulation of the B-cell receptors (BCR), NFkB, Notch (*see articles associated to this Frontiers topic*) or BCL2-family networks, leading to increased survival and enhanced chemoresistance.

BCL2-family proteins, which play a central role in the control of apoptosis, include multidomain anti-apoptotic members (Bcl-2, Bcl-xL, Mcl-1, Bcl-w, Bfl-1), BH3-only sensitizers (Bad, Bik, Noxa, Hrk, Bmf), BH3-only activators (Bid, Puma, Bim), and pro-apoptotic effectors (Bax, Bak) (6). The deregulation of the “B-cell lymphoma-2” (BCL2) family in mature B cell malignancies has been first highlighted through a translocation between the chromosomes 14 and 18 that led to the overexpression of the Bcl-2 oncogene in follicular lymphoma (7). Additional deregulations were then described such as 1q amplification leading to Mcl-1 overexpression in MM (8), Bim deletion in lymphoma cell lines (9) or miRNA deregulation leading to Bcl-2 overexpression in CLL (10, 11).

Given the central role of the BCL2-family in the apoptosis machinery, several strategies have been developed to target this network in hematological malignancies, such as synthetic antisense, specific peptides or BH3-mimetics (12, 13). Up to day, BH3-mimetics displayed the best efficacy both *in vitro* and *in vivo* (14, 15). Indeed, BH3-mimetics selectively bind anti-apoptotic members of the BCL2-family with high affinity, leading to the release of pro-apoptotic members that consequently induce cell death (16). Several clinical trials are currently ongoing using the first in class orally bioavailable BCL2-selective BH3-mimetic venetoclax, demonstrating clinical efficacy as a single agent in several B cell malignancies such as CLL, MCL, and MM (17–21).

Nevertheless, mature B cell neoplasms do not harbor similar dependence to anti-apoptotic members of the BCL2-family. For example, whereas both CLL and DLBCL overexpress Bcl-2 protein (10, 22), the overall response rate (ORR) of patients to venetoclax-monotherapy strongly diverged with 79 and 18%, respectively. In addition to intrinsic resistance, acquired resistance to BH3-mimetics has also been recently described (23–25). The challenge is now to set up markers and functional assays that predict responses to BCL2-family targeted strategies and to design mechanism-based combinations to overcome resistance.

To gain insight into BCL2-family expression and regulation across most frequent mature B cell malignancies, we analyzed the BCL2-family expression in ten different hematological disorders i.e., MCL, BL, DLBCL, FL, B-cell prolymphocytic leukemia (BPLL), CLL, HCL, mucosa-associated lymphoid tissue (MALT), SMZL, MM, through normalization of Affymetrix Human Genome U133 Plus 2.0 public datasets. We analyzed: (1) the common modulations across all B-cell neoplasms in comparison with their respective normal counterpart, (2) the modulations associated to the microenvironment and molecular subtypes, and (3) established a ratio of expression involving Bcl-2, Bcl-xL, Bax, and Bim that is associated with the response rate to venetoclax.

## Materials and Methods

Gene expression profiling datasets were selected on Gene Expression Omnibus (https://www-ncbi-nlm-nih-gov.gate2.inist.fr/geo/) and ArrayExpress (https://www.ebi.ac.uk/arrayexpress/), for all mature B-cell malignancies series and normal B-cell series (Table S1). In order to overcome data normalization biases, only Affymetrix Human Genome U133 Plus 2.0 series with raw data were retained. Raw data (cel files) were acquired as a whole and normalized using Affy and gcrma packages and outlier samples were removed and data were further quantile normalized (Figure S1A). Normalization quality and the absence of a remnant batch-effect were further assessed by the analysis of “anchoring genes” expression (*CD27, CCND1, SOX11, MKI67, BCL6, MME, CD200, ITGAE, CD38*, and *SDC1*), highlighting histological and/or B-cell differentiation specificities, independent of source series (Figure S1). Normal counterpart B-cell were associated to B cell malignancies according to cell-of-origin classification (26). For genes with multiple Affymetrix probes, probes were selected according to correlations between GEP and RNA-seq data for MM and MCL cell lines when available (https://www.keatslab.org/data-repository) (*n* = 19) (Table S2). Given that none of the *BAD* and *HRK* probes available gave a correlation with RNA-seq, these genes were excluded from our study. In addition, expression of *BBC3* (coding for Puma protein) has not been evaluated because of putative *MIR3191/MIR3190* cross-hybridization (Affymetrix HGU133plus2.0 Annotation, Revision 35).

Factor maps were constructed by FactoMiner and further represented by factoextra package. Data used in the Principal Component for each graph were a subset of the Bcl2-family dataset we firstly constructed.

For quantitative variables, statistical testing was performed using Wilcoxon-Mann-Whitney tests for two groups and Kruskal-Wallis for more than two groups. For qualitative variables, Fisher-test was performed. Statistical significance was retained under α-risk of 0.05. Random forest analysis was carried-out with 1,000 trees, using randomForest R-package.

## Results

### B-cell Malignancies Display Unbalanced Regulations of their Anti- and Pro-apoptotic Genes

**Figure 1:**
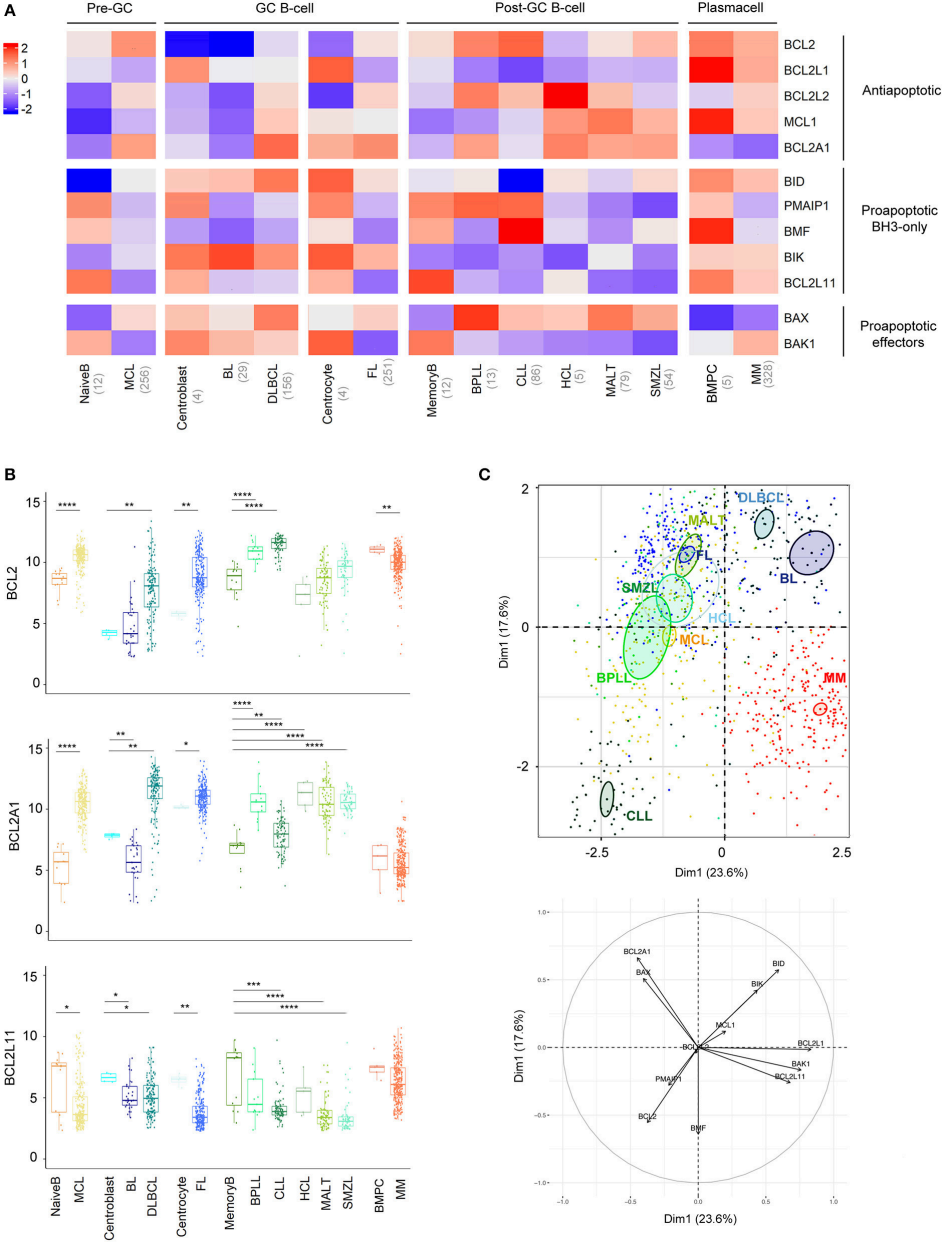
BCL2-family is strongly deregulated in the landscape of B-cell malignancies. **(A)** Heat-map of Bcl-2 gene expression profiles among B-cell malignancies. The color corresponds to the intensity of the median gene expression. Blue indicates lower and red higher transcript abundance. MCL, Mantle Cell Lymphoma; BL, Burkitt lymphoma; DLBCL, Diffuse Large B-cell Lymphoma; FL, Follicular Lymphoma; BPLL, B-cell Prolymphocytic Leukemia; CLL, Chronic lymphocytic leukemia; HCL, Hairy Cell Lymphoma; MALT, mucosa-associated lymphoid tissue lymphoma; SMZL, Splenic Marginal Zone Lymphoma; BMPC, Bone Marrow Plasma Cell, MM: multiple Myeloma. **(B)** Expression of *BCL2, BCL2A1*, and *BCL2L11* in the different B-cell malignancies compared to their respective control. Wilcoxon-Mann-Whitney tests. ^*^p < 0.05, ^**^p < 0.01, ^***^p < 0.001, ^***^*p < 0.0001. **(C)** Representation of the individual factor map of each sample for the PCA and according to the two first dimensions. Colored ellipses are drawn around the mean of the group (= barycenter), with the 95% confidence interval of the mean in the corresponding plan. *BCL2* is coding for Bcl-2 protein, *BCL2L1* for Bcl-xL, *MCL1* for Mcl-1, *BCL2L2* for Bcl-w, *BCL2A1* for Bfl1, *BIK* for Bik, *PMAIP1* for Noxa, *BMF* for Bmf, *BID* for Bid, *BCL2L11* for Bim, *BAX* for Bax, and *BAK1* for Bak.

B cell malignancies were classified and compared to their normal B cell counterparts according to the latest WHO classification (26). Whereas, MCL was defined as a pre-GC (germinal-center) neoplasm, FL, BL, and DLBCL were defined as GC neoplasms and SMZL, MALT, BPLL, CLL, HCL, and MM as post-GC neoplasms (Figure 1A). Within GC neoplasms, we further compared highly proliferative BL and DLBCL to centroblasts and the mostly indolent FL to centrocytes.

Anti-apoptotic members of the BCL2-family have a tendency to be overexpressed in most malignancies compared to their relative normal control, with the striking exception of *BCL2L1*, coding for BCLxL protein (Figure 1A, Figure S2). *BCL2* was overexpressed in MCL, DLBCL, FL, BPLL, and CLL. Of note, *BCL2A1*, coding for Bfl1 protein, appeared to be the most frequently elevated genes (8 out 10 malignancies, Figure 1B). As previously described, overexpression of *BCL2A1* was not observed in MM (27). Furthermore, in contrast to most mature B cell malignancies, MM and BL did not show major modulations of anti-apoptotic genes when compared to their normal counterparts (Figure 1A, Figure S2).

Pro-apoptotic BH3-only have a tendency to be downregulated in all mature B cell malignancies compared to their relative normal control, *BCL2L11*, coding for Bim protein, being the most frequently significantly deregulated gene (7 out of 10 malignancies, Figure 1B, Figure S2). Regarding pro-apoptotic effectors we observed a *BAX*/*BAK1* switch of expression in malignant B cells compared to their normal counterparts. Indeed, whereas *BAX* was elevated, *BAK1* appeared downregulated in all malignancies, excepted in MM and BL (Figure 1A, Figure S2).

To compare the 10 entities studied in regard to their BCL2-family profile, we performed a Principal Component Analysis (PCA, Figure 1C). We observed that CLL and MM displayed unique profiles. The variable plot highlighted that CLL profile was mostly carried by the expression of *BCL2, BMF, PMAIP1*, coding for Noxa protein, and the absence of *BID* whereas MM cells were characterized by the projection of *BCL2L1, BAK1*, and *BCL2L11* and the absence of *BCL2A1* (Figure 1C, lower panel).

### BCL2-family Genes Display Differential Expression According to the Microenvironment

**Figure 2:**
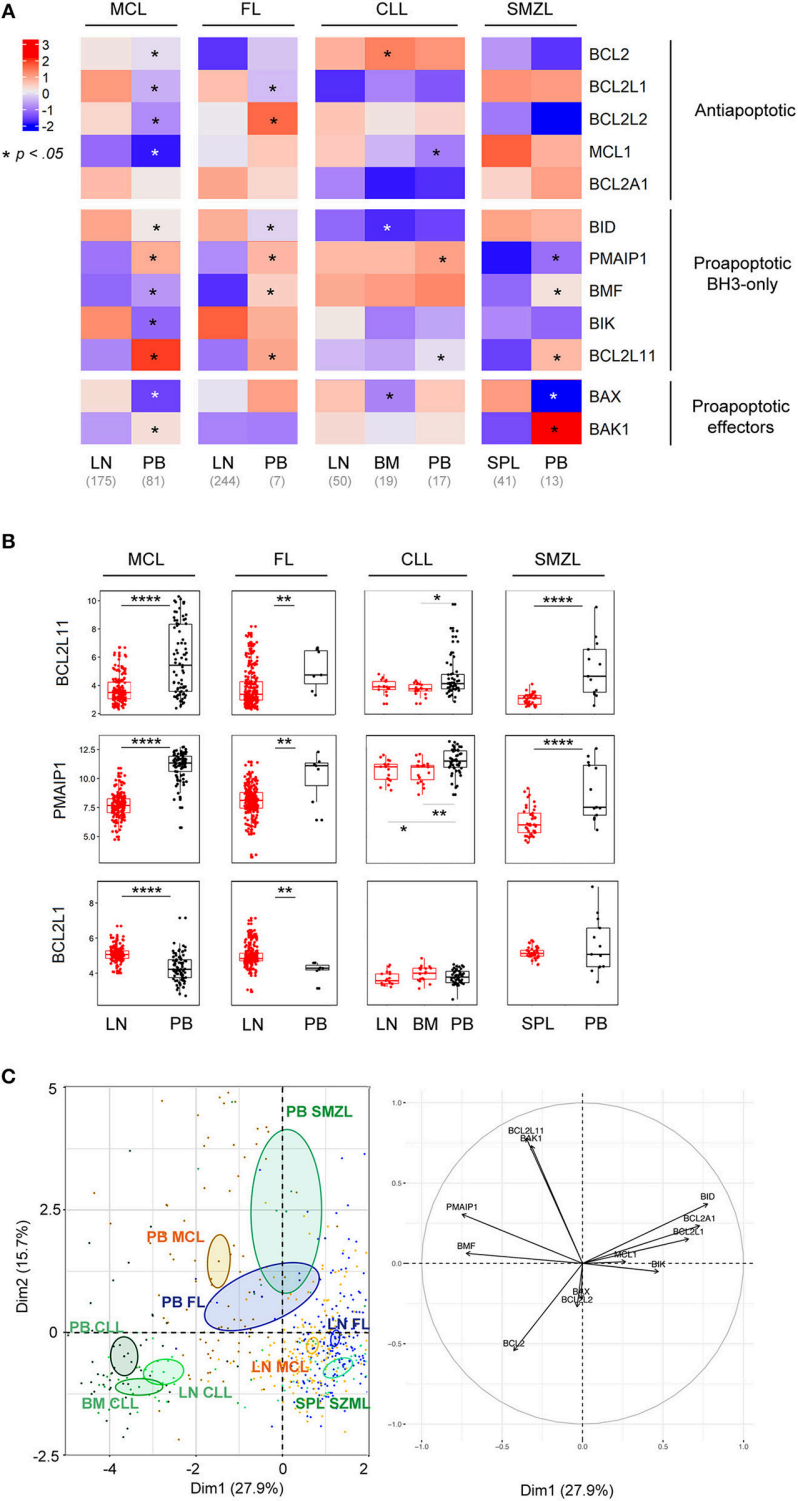
BCL2-family is regulated by the tumor microenvironment. **(A)** Heat-map of Bcl-2 gene expression profiles for MCL, FL, CLL, and SMZL in function of their tissue localization. Wilcoxon-Mann-Whitney tests. ^*^p < 0.05. **(B)** Comparison of *BCL2L11, PMAIP1*, and *BCL2L1* gene expression according to their localization. LN, lymph nodes; PB, peripheral blood; BM, bone marrow. Wilcoxon-Mann-Whitney tests. ^**^p < 0.01, ^****^p < 0.0001. **(C)** Representation of the individual factor map for the PCA according to the two first dimensions and their respective correlation circle. Colored ellipses are drawn around the mean of the group (= barycenter), with the 95% confidence interval of the mean in the corresponding plan.

We, and others, previously demonstrated that microenvironment-dependent modulations of BCL2-family members were involved in the survival and chemoresistance of B cell malignancies (23, 28, 29). To get insight into the role of the microenvironment in the BCL2-family regulation, we compared the expression profile of lymphoma cells from peripheral blood (PB) and tumoral niches i.e., lymph nodes (LN), bone marrow (BM) or spleen (SPL) for MCL, FL, CLL, and SMZL. MCL displayed the most frequent modulations with 11 out of 12 genes being significantly differently expressed between LN and PB with a general increase of all anti-apoptotic members within LN (Figure 2A, Figure S3). Although PB and LN samples were not paired, these data suggest that MCL cells have divergent BCL2 profiles depending on their microenvironment.

Of interest, our analysis highlighted that, independently of the nature of malignant B cells, the pro-apoptotic BH3-only *BCL2L11* and *PMAIP1* genes were deeply repressed in tumor niches (Figure 2B). In contrast, anti-apoptotic regulation seemed to be cell-type specific and only *BCL2L1* was commonly upregulated in the LN of both MCL and FL (Figures 2A,B).

PCA of these entities showed that tumor localization prevailed over entity intrinsic hallmarks (Figure 2C). Indeed, PB lymphoma cells from FL, MCL, and SMZL segregated together and apart from their relative LN cells. In contrast, CLL samples form a separated group independent of their tumor localization (PB, LN, and BM), confirming the specific profile of this malignancy as mentioned before (Figures 1C, 2C).

### Intra-entities BCL2-family Heterogeneity Is Related to Molecular Subtypes and Aggressiveness

**Figure 3:**
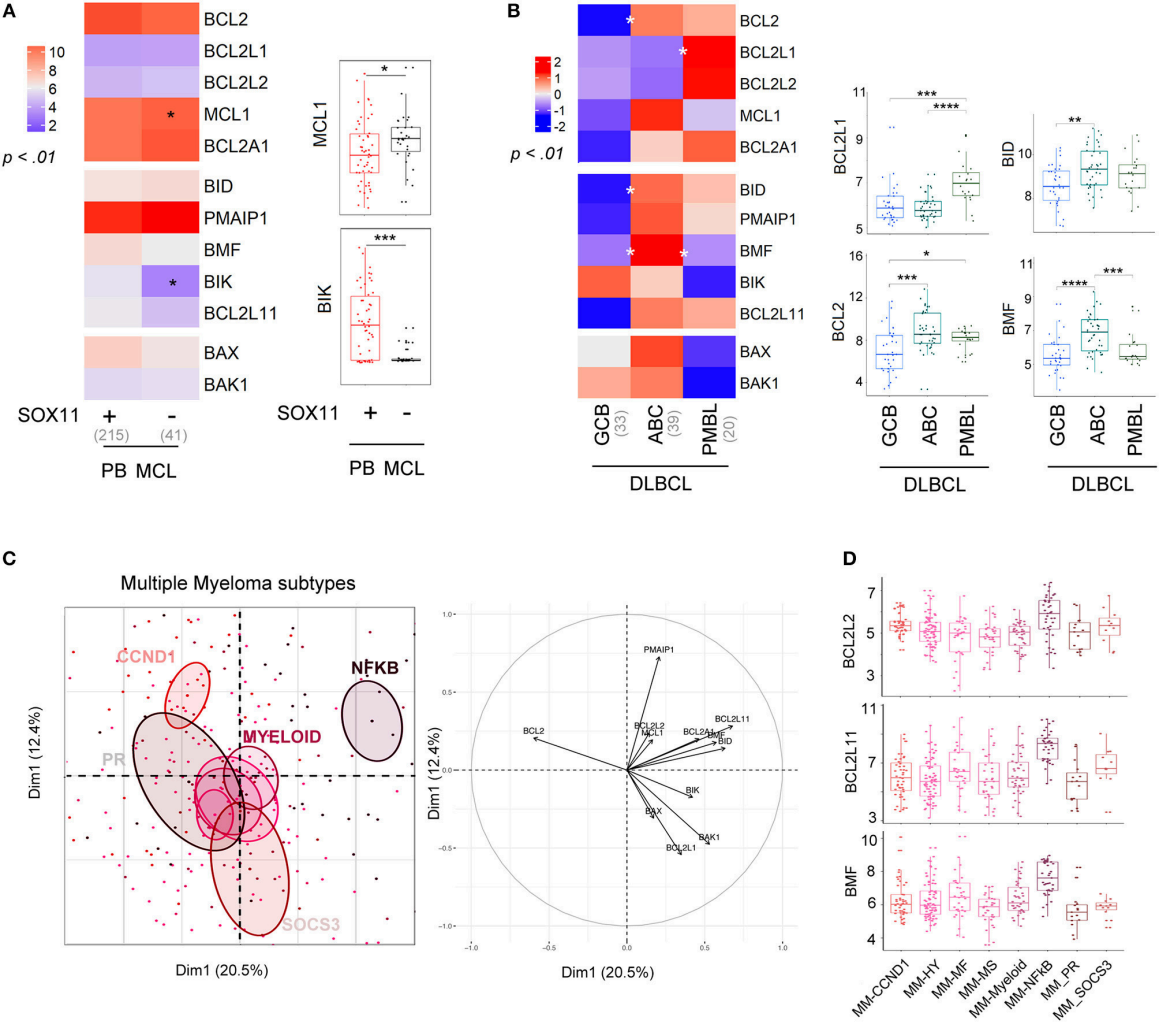
Molecular subtypes display differential expression of the BCL2-family. **(A)** left; Heat-map of BCL2-family expression profile comparing PB MCL according to *SOX11* gene expression (right). *MCL1* and *BIK* expression in the two molecular subgroups. **(B)** left; Heat-map of BCL2-family expression profile comparing the different subtypes of DLBCL: GCB (germinal center B cell, ABC (activated B-cell) and PMBL (Primary mediastinal B-cell lymphoma). right; Gene expression of *BCL2L1, BCL2, BID*, and *BMF* for the three subtypes of DLBCL. **(C)** Representation of the individual factor map for the PCA and according to the two first dimensions of multiple myeloma samples and their respective correlation circle. Colored ellipses are drawn around the mean of the group (= barycenter), with the 95% confidence interval of the mean in the corresponding plan. **(D)** *BCL2L2, BCL2L11*, and *BMF* expression in the different multiple myeloma subtypes. ^*^p < 0.05, ^**^p < 0.01, ^***^p < 0.001, ^****^p < 0.0001.

Molecular subgroups have been previously described in several B cell disorders (26). We thus compared the BCL2 profile according to molecular subtypes in DLBCL, MCL, and MM (Figure 3, Figures S4, S5).

Conventional MCL cells are characterized by a strong expression of the oncogene *SOX11*. A *SOX11*-negative (*SOX11-*) leukemic non-nodal minor MCL subtype is now well-characterized and displays a limited number of genomic alterations and a more indolent clinical course (30). The BCL2-family profile of conventional PB *SOX11*+ MCL was mostly similar to the one of leukemic non-nodal *SOX11-* MCL (Figure 3A). Nevertheless, *SOX11-* MCL cells displayed a moderate increase in MCL1 expression and a dramatic decrease in BIK expression when compared to *SOX11*+.

We next compared the profile of 3 subtypes of DLBCL, GC-type (GCB), ABC-type (ABC), and primary mediastinal (PMBL, Figure 3B). Our analysis showed that ABC cells were characterized by a high level of *BCL2, BID*, and *BMF*, which is consistent with previous reports (31). In contrast, PMBL cells displayed a high expression of *BCL2L1* (Figure 3B).

Several gene-expression profiling analyses of primary MM cells have led to a molecular classification of MM subtypes (32–34). This classification now includes 8 subgroups characterized either by an IgH translocation with the CyclinD1 [*t*_(11;14)_; CCND1 group], the MMSET oncogene [*t*_(4;14)_; MS group], MAF oncogenes [*t*_(14;16)_ and *t*_(14;20)_], or by specific gene signatures (PR, HY, Myeloid, SOCS3, and NFKB) (35, 36). We previously reported the apoptotic machinery diversity in MM major subgroups (HY, CCND1, MF, and MS) (37). Here, we enlarged the analysis by taking into account the 8 molecular subgroups (33). As represented by PCA, the NFKB subgroup displayed a specific BCL2-family profile and was characterized by an overexpression of *BCL2L2, BCL2L11*, and *BMF*, while the other groups overlapped without any exclusive signatures (Figures 3C,D).

**Figure 4:**
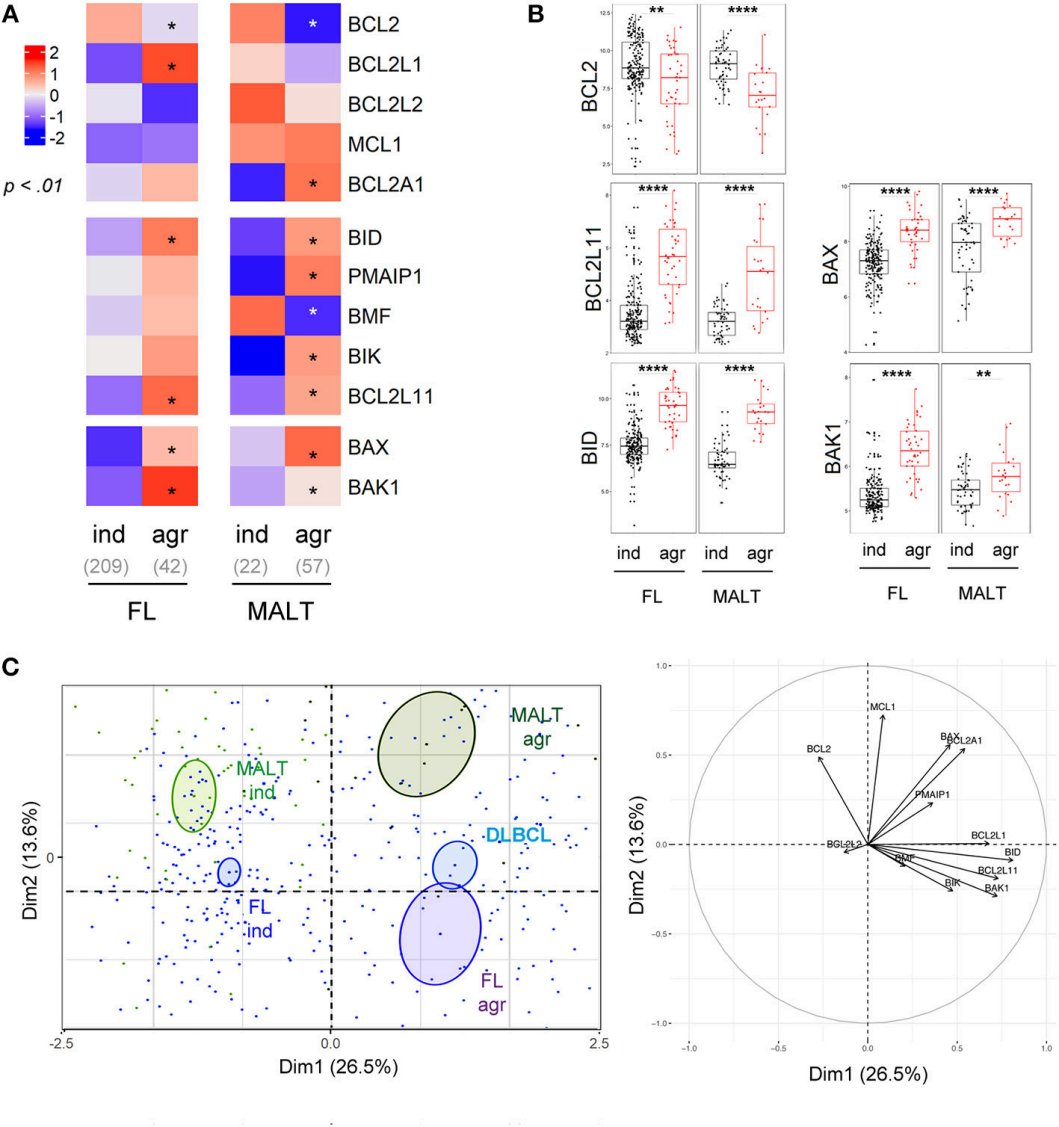
Intra-entities BCL2-family heterogeneity is related to aggressiveness. **(A)** Heat-map of BCL2-family expression profile comparing FL and MALT samples according to their indolent (ind) or aggressive (agr) status. **(B)** *BCL2, BCL2L11, BID, BAX*, and *BAK1* expression in FL and MALT sample according to their aggressiveness. **(C)** Representation of the individual factor map for the PCA and according to the two first dimensions of lymphoma samples and their respective correlation circle. Colored ellipses are drawn around the mean of the group (= barycenter), with the 95% confidence interval of the mean in the corresponding plan. ^*^p < 0.05, ^**^p < 0.01, ^****^p < 0.0001.

Histologic transformation of indolent B cell lymphomas such as FL or MALT into an aggressive lymphoma (mostly DLBCL) is a well-described phenomenon (38). Our analysis highlighted that histologic transformation was associated with common deregulations of the BCL2-family in both FL and MALT (Figure 4A, Figure S6). Indeed, we observed a downregulation of BCL2 as well as an increase of the pro-apoptotic *BCL2L11, BID*, and *BAX* and *BAK1* in both entities after transformation (Figure 4B). As observed in the PCA, the BCL2-family profile of the aggressive forms of both FL and MALT segregated apart from their respective indolent forms toward a profile close to the one of DLBCL (Figure 4C). Of note, we investigated whether BCL2-family expression patterns would differentiate the non-transformed FL/MALT from the transformed one. To do so, an ensemble machine-learning algorithm (random forest) was trained on BCL2-family expression dataset to predict the different B-cell malignancies. Using this trained algorithm on FL and MALT, it classified the transformed forms of the latters as DLBCL, thus efficiently predicting the aggressive transformation in both FL [Odds Ratio [OR] for transformation = 31, *p* = 2x10^−14^] and MALT (OR = 30, *p* = 9 × 10^−5^).

### BCL2-family Expression Profile Predicts the Sensitivity to BCL2 Specific BH3-mimetics in Mature B Cell Malignancies

**Figure 5:**
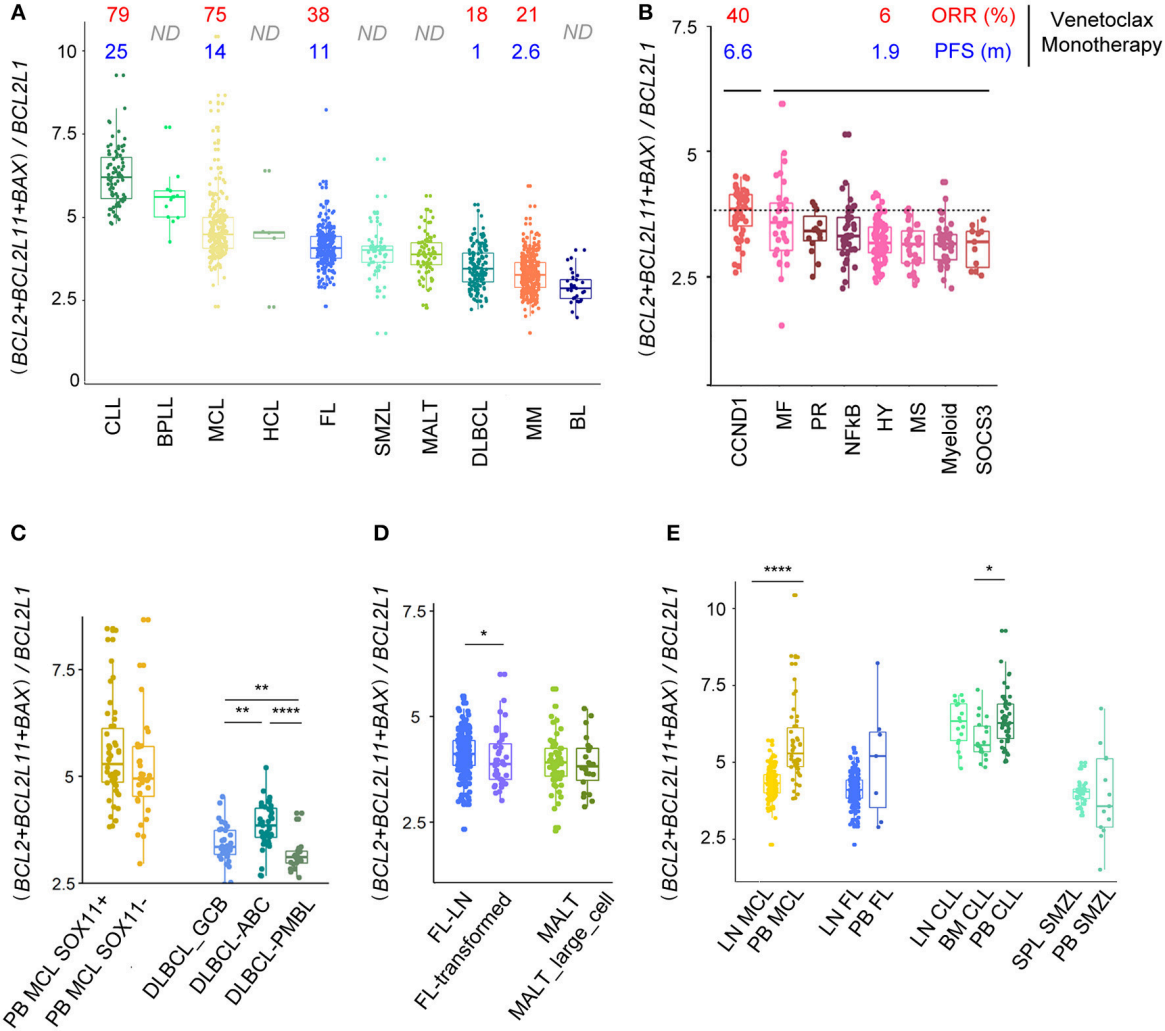
(*BCL2*+*BCL2L11*+*BAX*)/*BCL2L1* ratio predicts response to Bcl2 specific BH3 mimetic. **(A)** Evaluation of the (*BCL2*+*BCL2L11*+*BAX*)/*BCL2L1* ratio for the different B-cell malignancies associated to ORR and PFS of patients treated with venetoclax-monotherapy when available. **(B,C)** Evaluation of the (*BCL2* + *BCL2L11* + *BAX*)/*BCL2L1* ratio for the different subtypes of **(B)** MM and **(C)** MCL and DLBCL. **(D)** Evaluation of the (*BCL2* + *BCL2L11* + *BAX*)/*BCL2L1* ratio for the different subtypes of FL and MALT. **(E)** Evaluation of the (*BCL2* + *BCL2L11* + *BAX*)/*BCL2L1* ratio for MCL, FL, CLL, SMZL according to their tissue localization (peripheral blood, PB, lymph nodes, LN, spleen, SPL). ^*^p < 0.05, ^**^p < 0.01, ^****^p < 0.0001.

We previously demonstrated that a ratio of *BCL2* expression with the resistance factors *MCL1* and *BCL2L1* could predict sensibility to venetoclax in MCL and MM *ex vivo* and *in vivo* (20, 39, 40). Here, to determine the best predictive ratio across mature B cell malignancies, we analyzed the correlations between expression of previously described factors involved in venetoclax resistance (*MCL1, BCL2L1, BCL2A1*) (14, 23, 25, 39–41) as well as factors involved in venetoclax efficacy (*BCL2, BCL2L11, BAX*) (24, 25, 42) with overall response rate (ORR) in patients treated with venetoclax. Recent publications have shown an elevated ORR of venetoclax monotherapy in CLL and MCL (79 and 75%, respectively) (18, 20), intermediate for FL (38%) (17) and low for DLBCL and MM (18 and 21%, respectively) (17, 20). We showed that the ratio *(BCL2*+*BCL2L11*+*BAX)/(BCL2L1)* was the best predictor of venetoclax response across all mature B cell malignancies (*r* = 0.81, *p* = 7e-4, Figure S7). Of note, BPLL and HCL, entities for which venetoclax efficacy is unknown, were characterized by a high ratio whereas BL was characterized by a low ratio (Figure 5A).

We next analyzed whether subgroups of patients (genomic heterogeneity or transformation) displayed different ratios. In good agreement with the *in vivo* and *in vitro* sensitivity to venetoclax, we showed that the CCND1 MM subgroup displayed the highest *(BCL2*+*BCL2L11*+*BAX)/(BCL2L1)* ratio among MM subtypes (Figure 5B) (14, 20). Interestingly, subgroups of patients with MCL (*SOX11*+/–) harbored similar ratio, while ABC DLBCL cells were characterized by a higher ratio compared to GCB and PMBL. Histologic transformation only slightly influenced the ratio in FL but not in MALT lymphoma (Figures 5C,D).

Lastly, we compared the *(BCL2*+*BCL2L11*+*BAX)/(BCL2L1)* ratio according to the microenvironment and showed that MCL within the LN are predicted to be more resistant to venetoclax that MCL cells in the PB, confirming our previous functional *in vitro* observations (23, 39). Similarly, our analysis predicted that CLL cells should be less sensitive to venetoclax in BM as compared to PB (Figure 5E).

## Discussion

The BCL2-family is known to be deregulated in cancer, including hematological malignancies (43). Whereas, most studies focused on the regulation of selective BCL2-family members within a specific pathology, here we provided a global RNA expression analysis of 12 members of the BCL2-family across 10 mature B-cell malignancies and their relative normal counterparts. To do so, we took advantage of the numerous Affymetrix HGU133Plus2.0 series datasets previously published for mature B cell malignancies and gathered in the GEO database. We controlled the normalization quality by addressing hallmarks expression such as *CCND1, SOX11, MKI67, MME, CD200*, CD38, or *SDC1*, confirming malignancies specificities, independently of source series (Figure S1). Using similar data mining strategy, Adams et al. recently highlighted an overexpression of *BCL2* and *BCL2L2* in Hodgkin Lymphomas and several NHL (BL, DLBCL, FL, MZL, and MCL) (44). This overexpression was confirmed in our study with the exception of BL, a discrepancy that might be due to the use of different normal counterparts. Nevertheless, this technology has limitations such as probes aspecificity (*HRK, BAD*) or cross-hybridization within some probes such as *BBC3* (45), impeding the integration of these critical member of the BCL2 network in the present study (see Material and Methods section). Although this drawback could be resolved using RNA-sequencing technologies, datasets availability was too limited for most of the cellular entities analyzed in the present work.

Having these limitations in mind, our analysis provided a global picture of the BCL2-family dysregulation in mature B-cell malignancies, from their transcriptional regulation to their potential use as targeted therapy biomarker. We first highlighted a global upregulation of anti-apoptotic genes as well as a global downregulation of pro-apoptotic genes in most B cell lymphomas compared to their normal control, confirming that the BCL2-family deregulation is a hallmark of most B cell malignancies. We did not observe upregulation of the anti-apoptotic genes in MM compared to BMPC. On the one hand, this might be due to the elevated level of anti-apoptotic genes in BMPC, which are necessary for the survival of these long-lived cells (46). On the other hand, we cannot exclude that posttranscriptional modifications could directly influence protein levels, particularly for Mcl-1 (47–49).

We also showed specific modulations in BCL2-family expression associated to molecular subgroups in MCL, DLBCL and MM. In the *SOX11-* MCL subtype, we highlighted a selective dramatic downregulation of *BIK*. Given that this BH3-only is tightly regulated by DNA methylation (50), its silencing might be the direct consequence of the specific epigenetic profile recently described in this MCL subtype (51). Further investigations are now needed to document the consequences of these modulations in the survival and chemoresistance of *SOX11-* MCL cells. Similarly, the “NFkB” molecular subgroup displayed a unique BCL2-family profile within MM samples, highlighted by the overexpression of *BCL2L2, BMF*, and *BCL2L11*. Given that this subgroup is characterized by an elevated expression of NFkB targets, it is tempting to speculate that the NFkB pathway regulates these genes in MM, as it has been previously described for *BCL2L2* in B cell lymphoma (52). Nevertheless, the “NFkB” entity represents < 10% of the disease and the lack of relevant *in vitro* models for this molecular subgroup makes its study challenging (53).

By evaluating BCL2-family expression according to tissue localization, we observed a strong microenvironment-dependent regulation, especially in MCL and FL. Several studies have demonstrated the critical role of the microenvironment in the expansion and the chemoresistance of these hematological malignancies (54–56). Furthermore, we recently showed that a microenvironment-dependent upregulation of *BCL2L1* and downregulation of *BCL2L11* was involved in MCL chemoresistance (23). Of interest, a global pro- and anti- apoptotic imbalance was confirmed here in MCL. In addition, we showed that both *BCL2L11* and *PMAIP1* were downregulated by the tumor microenvironment in all the B-cell malignancies studied (MCL, FL, CLL, and SMZL), suggesting a fundamental role of these 2 specific BH3-only proteins in the microenvironment-dependent survival of lymphoma cells. Rational strategies to counteract their downregulation could then be critical to target lymphoma cells within the protective niches.

This global tissue-specific modulation in the BCL2 profile also directly impacted the predictive ratio to venetoclax sensitivity in MCL. Indeed, the *(BCL2*+*BCL2L11*+*BAX)/BCL2L1* ratio was much lower in LN-MCL samples compared to PB-MCL. Even though clinical studies highlighted an encouraging ORR in MCL patients treated by venetoclax monotherapy, the PFS observed appeared much lower than in CLL. Our study suggested that MCL cells in the LN could be more resistant to venetoclax than PB-MCL and consequently could be involved in the rapid relapse observed in this pathology. Strategies targeting the microenvironment in association with venetoclax could then increase treatment efficacy and delay relapse. We recently show that MCL primary cells egressing in the PB through BTK inhibition have a *BCL2* high/*BCL2L1* low profile and were highly sensitive to venetoclax (39). Similarly, we showed that microenvironment-dependent *BCL2L1* induction was counteracted with the anti-CD20 antibody obinutuzumab, leading to an increased venetoclax efficacy *ex vivo* (23). Similar results showing the benefit of targeting microenvironmental interactions to potentiate BH3-mimetics efficacy have been published in other B cell malignancies such as CLL and MM (28, 29).

Of note, the above-mentioned predictive ratio highlighted that previously untested entities in venetoclax clinical trials, especially B-PLL and HCL, have sensitive-like BCL2-family profile, suggesting that they should be included in future clinical trials. Lastly, given the heterogeneity among entities (molecular subgroups, aggressiveness, tissue), this ratio could help predicting the B cell lymphoma patients who would benefit to BCL2 specific BH3-mimetic based therapy.

## Author Contributions

BT and AP designed the project, performed bioinformatics analyses, and wrote the paper. CB participated in the bioinformatics analyses. PG-B, MA, and CP-D participated in the design of the study and in the writing of the article. DC designed the project and wrote the paper.

### Conflict of Interest Statement

The authors declare that the research was conducted in the absence of any commercial or financial relationships that could be construed as a potential conflict of interest.
