# Supplementary material for: BCL2-Family Dysregulation in B-Cell Malignancies: From Gene Expression Regulation to a Targeted Therapy Biomarker
Source: Front Oncol. 2019 Jan 7;8:645. doi: 10.3389/fonc.2018.00645 (PMC6330761; doi:10.3389/fonc.2018.00645)
Supplement: Supplementary file 1 [file Data_Sheet_1.PDF]

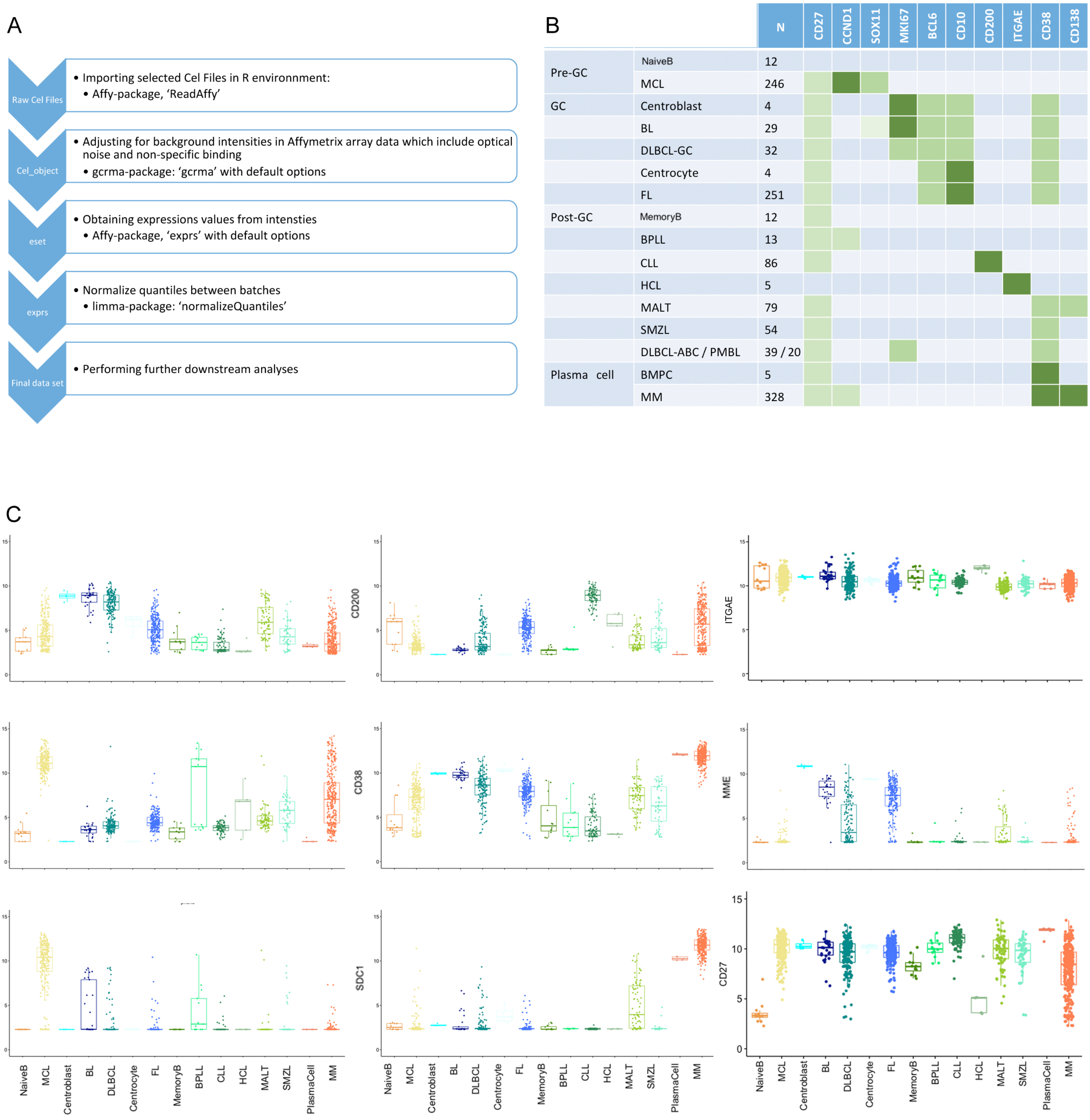

Figure S1. Work-flow and quality control. A. Flow diagram representing the work flow used in the study. B,C. Expression of "anchoring genes" (*CD27, CCND1, SOX11, MKI67, BCL6, CD10, CD200, CD38 and SDC1*) differencially expressed among the cell types analyzed.

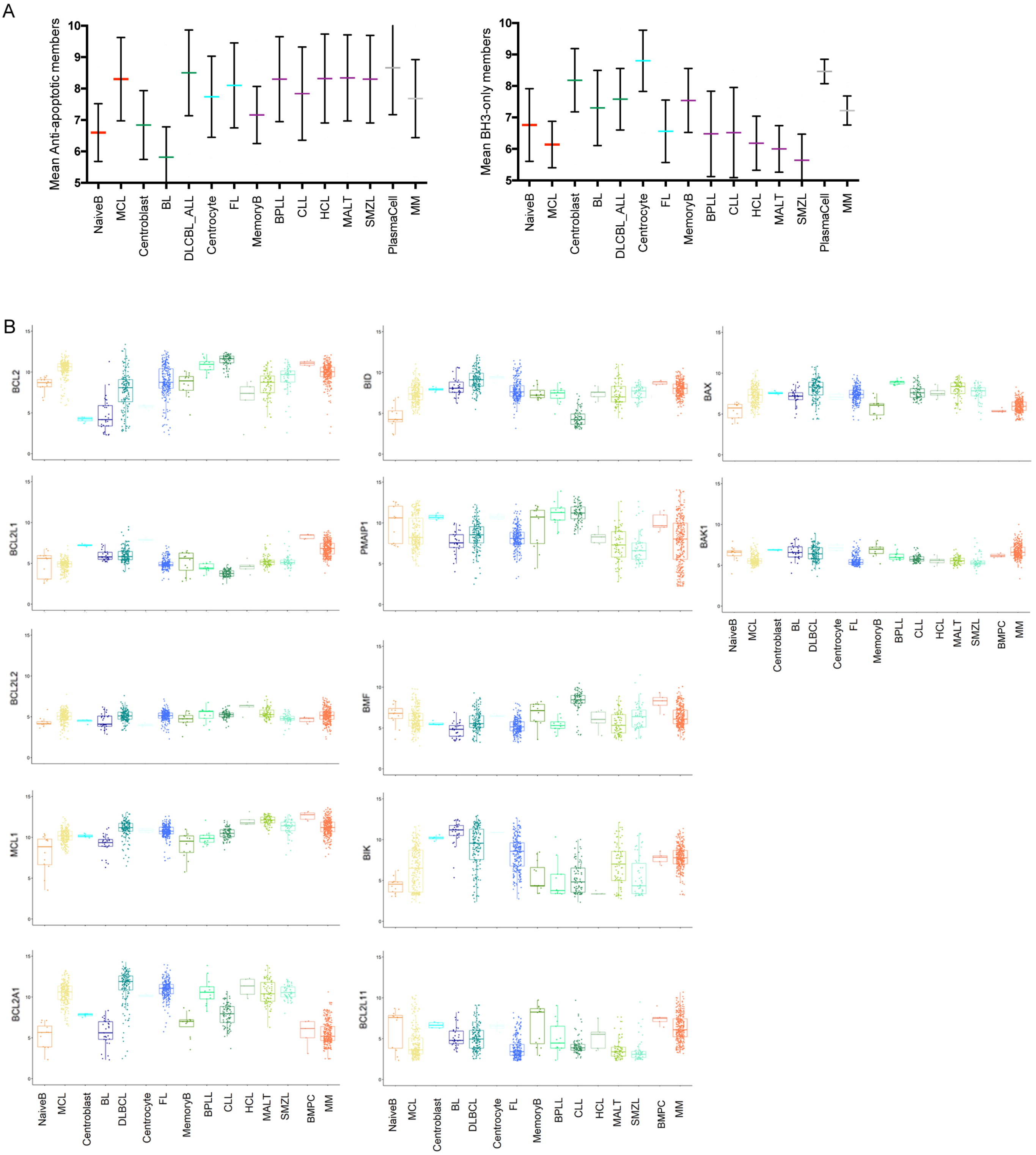

Figure S2. Expression of BCL2-genes family in the different B-cell malignancies compared to their respective control

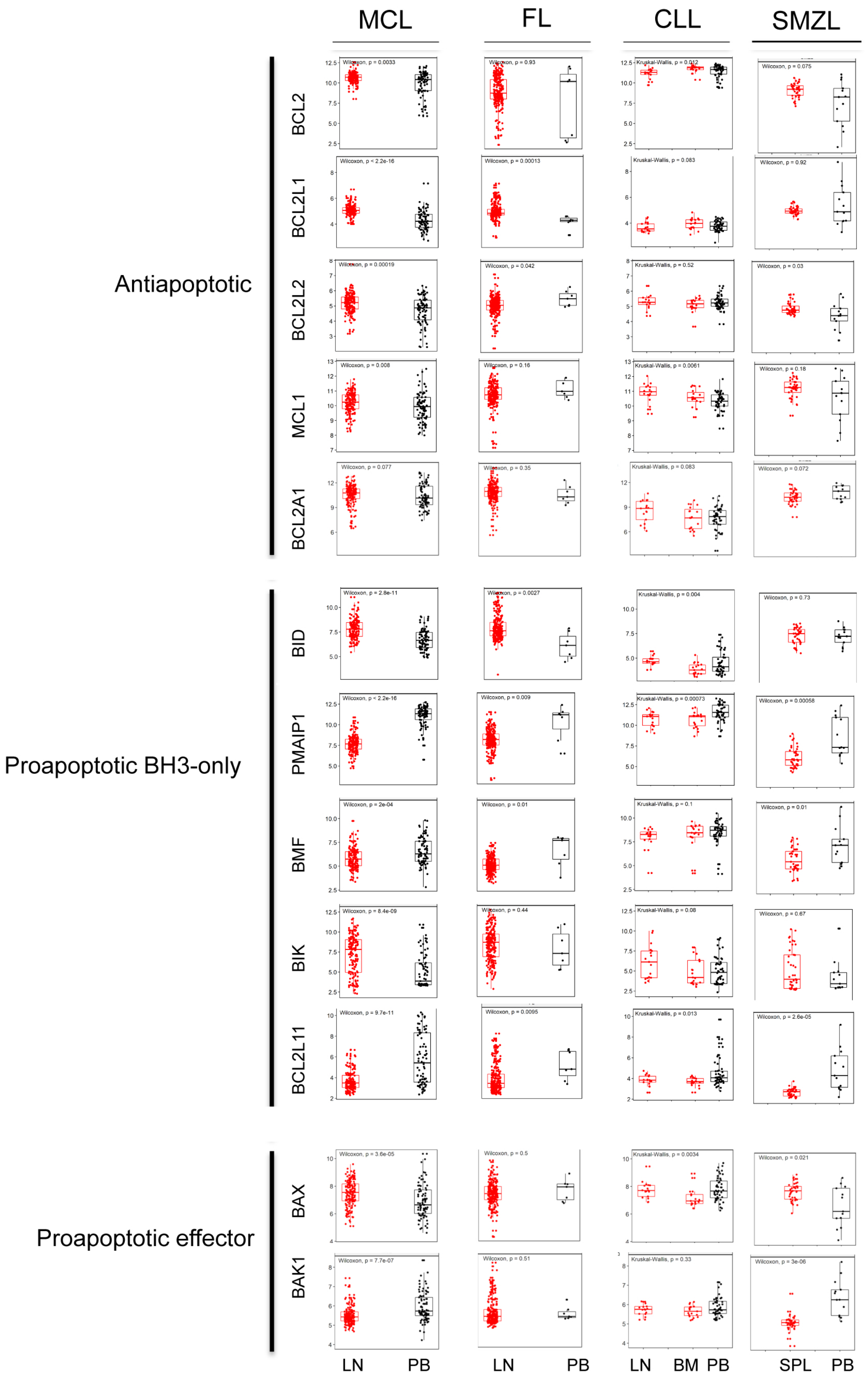

Figure S3. Expression of BCL2-genes family according to their localization. LN : lymph nodes, PB : peripheral blood, BM : bone marrow. Wilcoxon-Mann-Whitney tests or kruskal-Wallis tests.

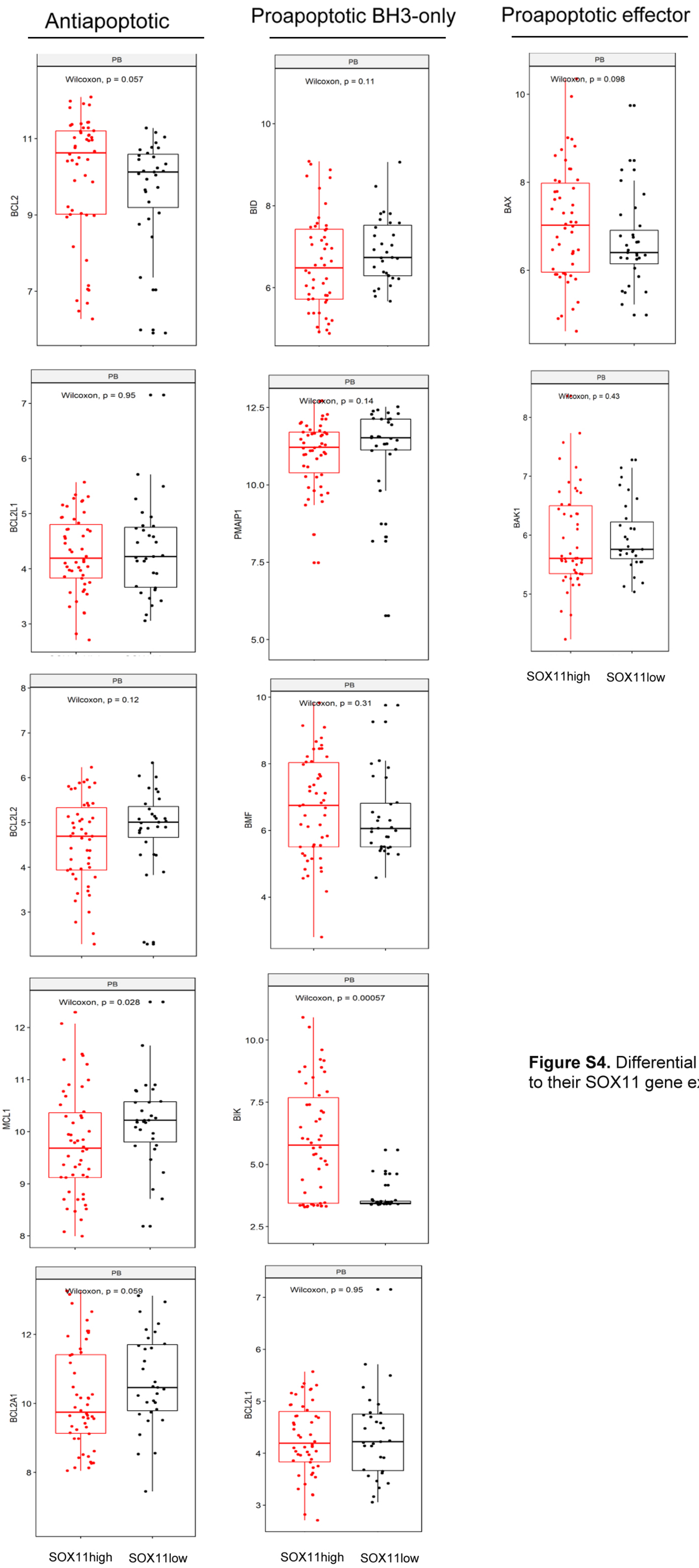

Figure S4. Differential expression of BCL2 family genes according to their SOX11 gene expression. Wilcoxon-Mann-Whitney tests.

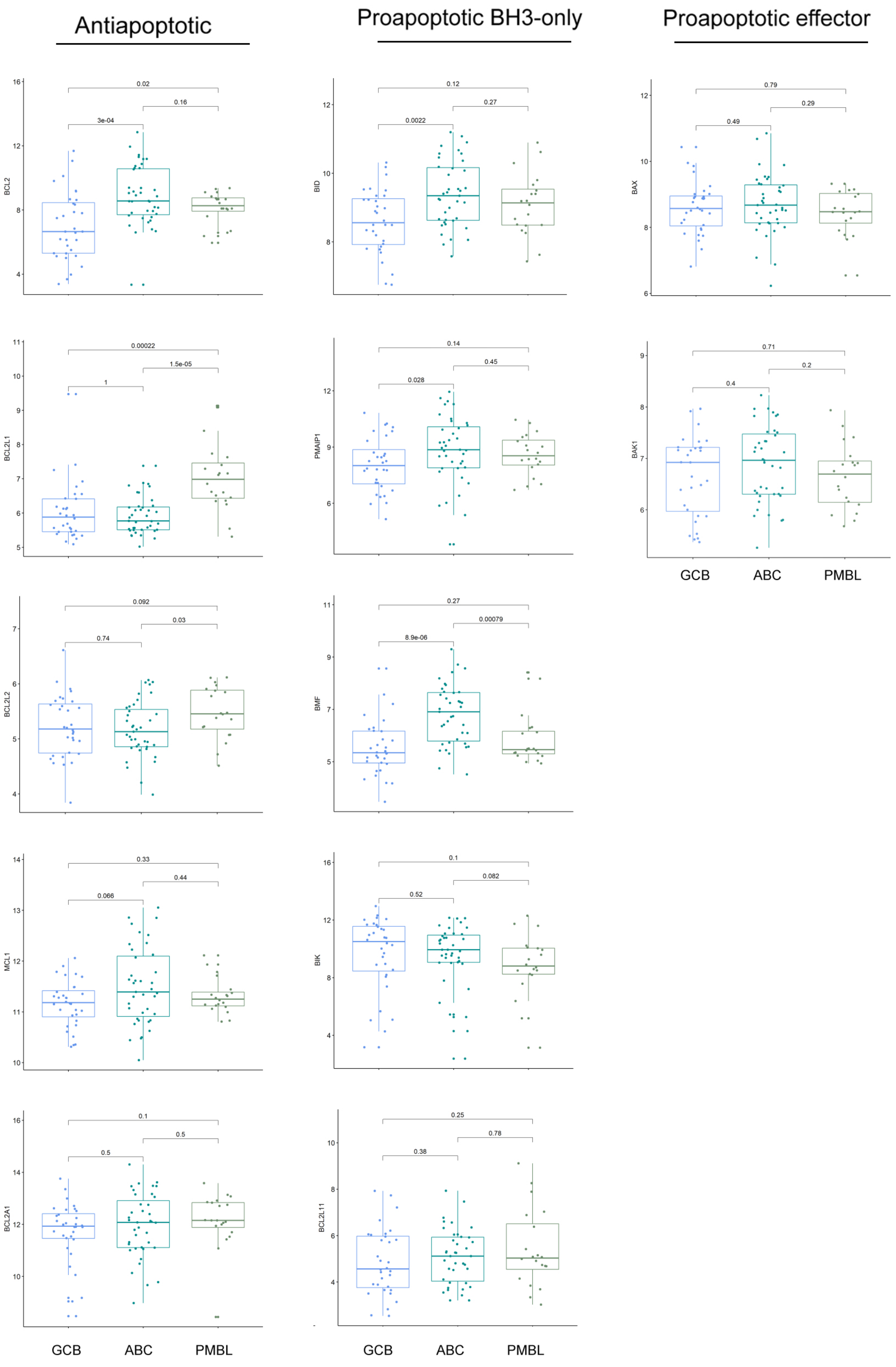

Figure S5. Gene expression of BCL2 family genes for the three subtypes of DLBCL: GCB (germinal center B cell), ABC (activated B-cell) and PMBL (Primary mediastinal B-cell lymphoma). kruskal-Wallis tests.

Antiapoptotic

Proapoptotic BH3-only

Proapoptotic effector

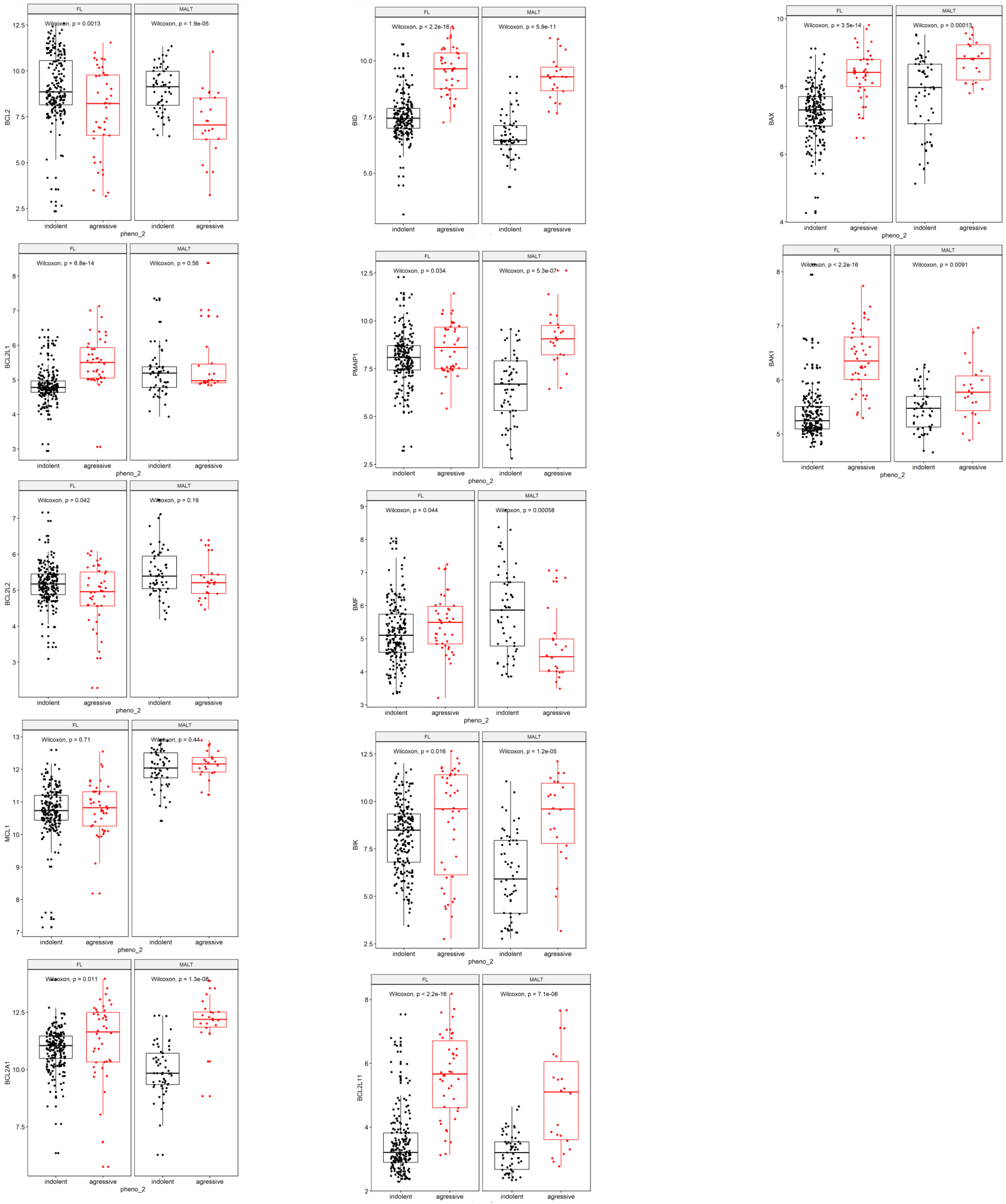

Figure S6. Gene expression of BCL2 family genes in FL (Follicular Lymphoma) and MALT (mucosa-associated lymphoid tissue lymphoma). Wilcoxon-Mann-Whitney tests.

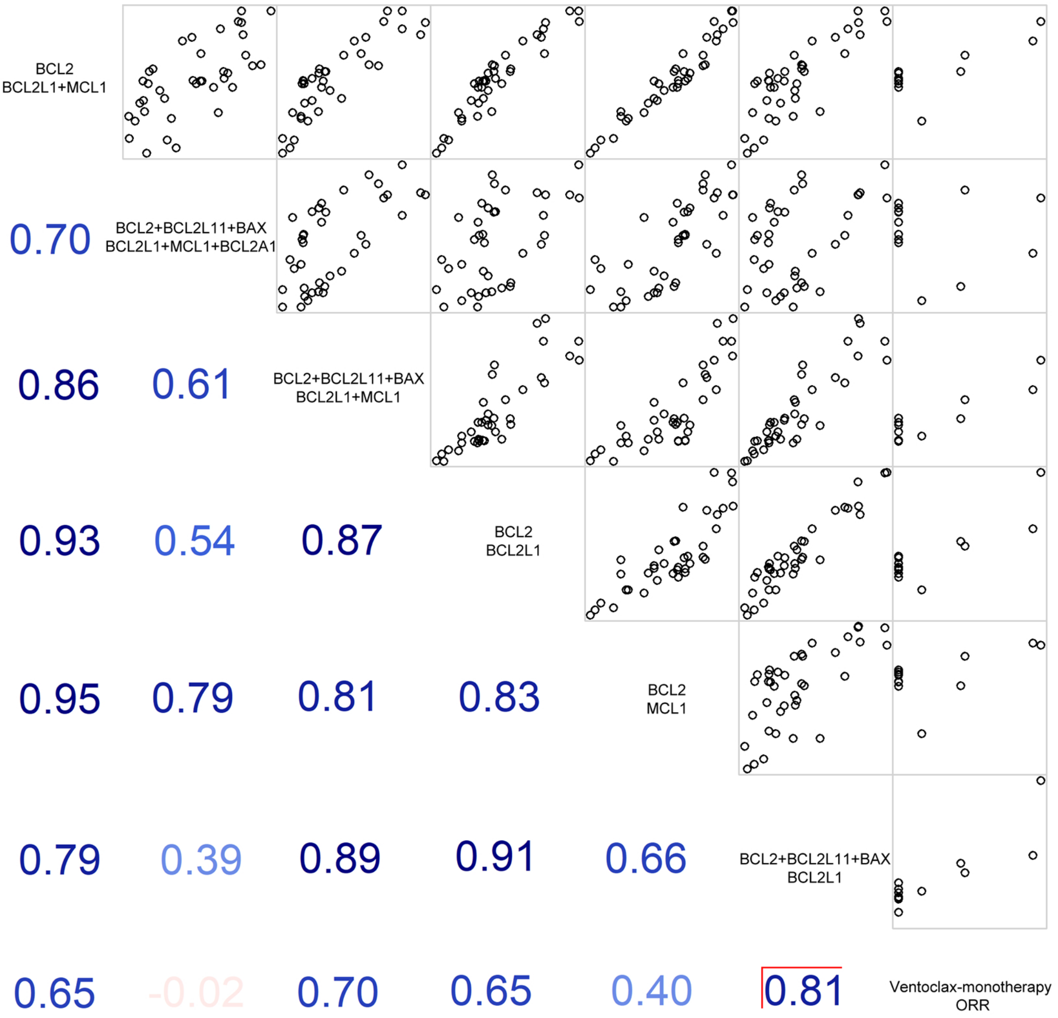

Figure S7. Correlations analysis between expression of factors involved in venetoclax resistance (MCL1, BCL2L1, BCL2A1) and factors involved in venetoclax efficacy (BCL2, BCL2L1, BAX) with venetoclax overall response rate (ORR).
